# Supplementary material for: Ladder‐Like Structural Architecture of Layered Magnetic A 2.4 Cr8Te14 (A = Rb, Cs) Compounds by Self‐Flux Synthesis
Source: Chemistry. 2026 Apr 10;32(24):e70897. doi: 10.1002/chem.70897 (PMC13290425; doi:10.1002/chem.70897)
Supplement: Supplementary file 1 — Supporting File: chem70897‐sup‐0001‐SuppMat.pdf. [file CHEM-32-e70897-s001.pdf]

Supplementary Information - Ladder-like Structural Architecture in Layered Magnetic $A_{2.4}Cr_8Te_{14}$ (A = Rb, Cs) Compounds by Self-flux Synthesis

Kai D. Röseler, Felix Eder, and Fabian O. von Rohr*

Department of Quantum Matter Physics, University of Geneva, CH-1211 Geneva, Switzerland

E-mail: fabian.vonrohr@unige.ch

Additional crystallographic comments

Structure determination and refinement of SXRD data was complicated by multiple factors, many of them linked to the layered character of $A_{2.4}Cr_8Te_{14}$. (A = Rb, Cs)

i) Many crystal plates of suitable size were bent or deformed easily when attempting to cut them with a surgical knife. ii) The layered structural architecture makes the crystal structures susceptible to stacking disorder. One possible defect concerns a translation of neighboring layers by $\mathbf{a}/3$ or $-\mathbf{a}/3$. As the outside edge of the Cr_8Te_{14} ladders has (in a simplified view) three times higher translational symmetry than the whole ladder, stacking errors appear likely. Another possible source of stacking faults concerns the alternation of disordered A layers and Cr_2Te_2 bridges + ordered A cations. Since the normal distance between two neighboring Cr_8Te_{14} ladders is almost the same as the distance between the two $CrTe_2$ layers forming a ladder (6.32 and 6.26 Å for $Cs_{2.4}Cr_8Te_{14}$, 6.12 and 6.15 Å for $Rb_{2.4}Cr_8Te_{14}$), stacking faults mixing up the two structural motifs in presented in Fig. 1 of the main article can easily be visualized. iii) The A sites in the layer interspace all exhibit significant under-occupation and therefore leave a lot of room for positional disorder. iv) Frequently, pieces of not fully removed flux were stuck on the crystallites. As the platelets were very susceptible to mechanical deformation, in some cases, small crumbles of flux remained on the crystals in order to not apply any mechanical stress to the crystal plates.

Choice of space group

Other than the $A_xCr_5Te_8$ phases, which are all found to crystallize in the $C2/m$ space group, the crystal structures of $A_{2.4}Cr_8Te_{14}$ follow the non-centrosymmetric Cm space group instead. The reason for this lies in the partially occupied layers of A cations intercalating the Cr_8Te_{14} ladders. For these $A2$, $A3$ and $A4$ sites, the $C2/m$ symmetry would result in unrealistically close A—A distances of ca. 2.25 Å. In Cm, all A sites are displaced by at least ca. 3.7 Å, and no significant difference electronic density is found on the poten-

Table 1: Crystallographic sites and their coordinates in the refinement of $Cs_{2.4}Cr_8Te_{14}$

Site	Wyckoff	Site symmetry	S.O.F	x	y	z
Cs1	$2a$	m	1	0.0002(3)	0	0.5002(3)
Cs2	$2a$	m	0.558(14)	0.0473(4)	0	0.0001(3)
Cs3	$2a$	m	0.363(14)	0.3907(7)	0	0.0008(4)
Cs4	$2a$	m	0.475(14)	0.7259(5)	0	0.0007(3)
Te1	$2a$	m	1	0.2199(2)	0	0.1682(2)
Te2	$2a$	m	1	0.5568(2)	0	0.1729(2)
Te3	$2a$	m	1	0.8874(3)	0	0.1688(2)
Te4	$2a$	m	1	0.1628(2)	0	0.3341(2)
Te5	$2a$	m	1	0.4951(3)	0	0.3333(2)
Te6	$2a$	m	1	0.8341(2)	0	0.3334(2)
Te7	$2a$	m	1	0.3292(3)	0	0.4970(2)
Te8	$2a$	m	1	0.6716(2)	0	0.5035(2)
Te9	$2a$	m	1	0.1677(2)	0	0.6687(2)
Te10	$2a$	m	1	0.5059(3)	0	0.6687(2)
Te11	$2a$	m	1	0.8379(3)	0	0.6672(2)
Te12	$2a$	m	1	0.1120(2)	0	0.8336(2)
Te13	$2a$	m	1	0.4438(2)	0	0.8300(2)
Te14	$2a$	m	1	0.7801(2)	0	0.8340(2)
Cr1	$2a$	m	1	0.0239(5)	0	0.2523(5)
Cr2	$2a$	m	1	0.3583(6)	0	0.2495(6)
Cr3	$2a$	m	1	0.6961(5)	0	0.2507(5)
Cr4	$2a$	m	1	0.7484(6)	0	0.4156(6)
Cr5	$2a$	m	1	0.2523(6)	0	0.5864(6)
Cr6	$2a$	m	1	0.3060(5)	0	0.7508(5)
Cr7	$2a$	m	1	0.6435(5)	0	0.7535(6)
Cr8	$2a$	m	1	0.9768(5)	0	0.7501(5)

Table 2: Crystallographic sites and their coordinates in the refinement of $Rb_{2.4}Cr_8Te_{14}$

Site	Wyckoff	Site symmetry	S.O.F	x	y	z
Rb1	$2a$	m	1	0.0003(4)	0	0.5004(4)
Rb2	$2a$	m	0.566(13)	0.0580(3)	0	0.0015(3)
Rb3	$2a$	m	0.342(14)	0.3784(7)	0	-0.0017(5)
Rb4	$2a$	m	0.490(14)	0.7232(4)	0	0.0010(3)
Te1	$2a$	m	1	0.22350(13)	0	0.17021(14)
Te2	$2a$	m	1	0.55530(15)	0	0.16297(16)
Te3	$2a$	m	1	0.88853(13)	0	0.16515(16)
Te4	$2a$	m	1	0.05396(15)	0	0.33185(16)
Te5	$2a$	m	1	0.39507(16)	0	0.33330(17)
Te6	$2a$	m	1	0.72566(18)	0	0.33250(18)
Te7	$2a$	m	1	0.33166(17)	0	0.50340(17)
Te8	$2a$	m	1	0.66815(16)	0	0.49634(16)
Te9	$2a$	m	1	0.27299(18)	0	0.66704(17)
Te10	$2a$	m	1	0.60364(16)	0	0.66583(16)
Te11	$2a$	m	1	0.94515(14)	0	0.66747(15)
Te12	$2a$	m	1	0.10837(13)	0	0.83465(16)
Te13	$2a$	m	1	0.44164(15)	0	0.83635(16)
Te14	$2a$	m	1	0.77370(14)	0	0.82924(14)
Cr1	$2a$	m	1	0.1363(3)	0	0.2476(4)
Cr2	$2a$	m	1	0.4735(3)	0	0.2460(4)
Cr3	$2a$	m	1	0.8109(3)	0	0.2514(3)
Cr4	$2a$	m	1	0.1950(3)	0	0.4142(4)
Cr5	$2a$	m	1	0.8053(4)	0	0.5854(4)
Cr6	$2a$	m	1	0.1875(3)	0	0.7488(3)
Cr7	$2a$	m	1	0.5242(3)	0	0.7531(4)
Cr8	$2a$	m	1	0.8627(3)	0	0.7518(4)

tial alternate A sites connected by the lost inversion symmetry. Nevertheless, the crystal structures of $Cs_{2.4}Cr_8Te_{14}$ and $Rb_{2.4}Cr_8Te_{14}$ needed to be refined as inversion twins. As the remainder of the crystal structure complies with $C2/m$ symmetry, this leads to correlations between refined parameters, manifesting in very anisotropic and sometimes even negative ADPs for most atomic positions. Therefore, the ADPs of the Cr and Te atoms were constrained to shared parameters for the pairs of atoms, which would correspond to the same crystallographic site in $C2/m$, using the EADP command in ShelXL. Doing so, only slightly increased the R-value of the refinement by roughly 0.001. These correlations are likewise the reason for the comparably high standard uncertainties of the atomic positions (Table 1,2) and consequently the interatomic distances (see Table 4) and angles in the refinement in Cm, they would be signifcantly smaller in $C2/m$.

Non-isotypic relation

To further underline the non-isotypic relation between the crystal structures of $Cs_{2.4}Cr_8Te_{14}$ and $Rb_{2.4}Cr_8Te_{14}$, the respective structural solutions were compared using the *compstru* software available at the Bilbao Crystallographic Server.[1] Hereby, one attempts to transform the second input crystal structure into the first one with minimum shifts of atomic positions. While the unit cells are similar with a degree of lattice distortion S of just 0.0089, the distance between paired atoms underlines the two different structure types. Interestingly, the sites belonging to the disordered A layer and the adjacent Te atoms (A2–4, Te1–3, Te12–14) have only marginal distances between corresponding paired atoms of 0.030–0.229 Å. However, the other atomic positions show very uniformous, high difference distances of 2.004–2.282 Å. These big differences result in a high measure of similarity δ – 0.379 (a low value close to 0 would indicate an isotypic relationship), thus confirming the non-isotypic relation between $Cs_{2.4}Cr_8Te_{14}$ and $Rb_{2.4}Cr_8Te_{14}$.

Table 3: Selected interatomic distances in the crystal structures of $Cs_{2.4}Cr_8Te_{14}$ and $Rb_{2.4}Cr_8Te_{14}$

	$Cs_{2.4}Cr_8Te_{14}$		$Rb_{2.4}Cr_8Te_{14}$	
Atom1	Atom2	distance / Å	Atom2	distance / Å
Cr1—	Te3	2.711(11)	Te1	2.707(7)
	Te5 (×2)	2.714(7)	Te2 (×2)	2.709(5)
	Te2 (×2)	2.728(7)	Te4	2.732(6)
	Te4	2.742(10)	Te6 (×2)	2.781(5)
Cr2—	Te3 (×2)	2.711(8)	Te2	2.704(7)
	Te6 (×2)	2.715(7)	Te3 (×2)	2.713(5)
	Te5	2.717(11)	Te4 (×2)	2.716(4)
	Te4	2.73(13)	Te5	2.719(7)
Cr3—	Te1 (×2)	2.693(7)	Te3	2.689(8)
	Te2	2.730(11)	Te5 (×2)	2.714(4)
	Te6	2.731(10)	Te6	2.730(8)
	Te4 (×2)	2.784(7)	Te1 (×2)	2.737(5)
Cr4—	Te8	2.687(12)	Te7	2.691(7)
	Te7 (×2)	2.716(8)	Te8 (×2)	2.714(5)
	Te6	2.740(12)	Te4	2.728(7)
	Te4 (×2)	2.757(9)	Te6 (×2)	2.740(5)
Cr5—	Te7	2.714(12)	Te8	2.697(7)
	Te9	2.720(12)	Te7 (×2)	2.708(5)
	Te8 (×2)	2.727(9)	Te11	2.708(7)
	Te11 (×2)	2.752(9)	Te9 (×2)	2.753(5)
Cr6—	Te13	2.711(12)	Te12	2.706(8)
	Te14 (×2)	2.719(7)	Te10 (×2)	2.720(4)
	Te9	2.733(10)	Te14 (×2)	2.722(5)
	Te11 (×2)	2.774(7)	Te9	2.745(8)
Cr7—	Te14	2.697(13)	Te11 (×2)	2.704(4)
	Te12 (×2)	2.724(8)	Te12 (×2)	2.711(5)
	Te9 (×2)	2.728(7)	Te13	2.720(7)
	Te10	2.736(11)	Te10	2.734(7)
Cr8—	Te12	2.689(11)	Te13 (×2)	2.694(5)
	Te10 (×2)	2.721(7)	Te11	2.736(6)
	Te13 (×2)	2.735(7)	Te14	2.737(7)
	Te11	2.749(10)	Te9 (×2)	2.783(5)
Cr4—	Cr3	3.087(15)	Cr1	3.065(10)
Cr5—	Cr6	3.078(15)	Cr8	3.061(10)

$Cs_{2.4}Cr_8Te_{14}$

For $Cs_{2.4}Cr_8Te_{14}$, diffuse streaks were clearly visible on the "superstructure" reflections corresponding to the triplication of **a**. (Figure 1) This fits the first suggested type of stacking disorder before in point ii). The "ladder" part consisting of the *A*1, Cr4, Cr5, Te7 and Te8 sites is the only section of the crystal structure that does not follow an idealized translational symmetry of **a**/3. Consequently, the most intense peaks of difference electronic density in the refinement correspond to the alternate Cr4 and Cr5 positions in this area. The alternate sites for Cs1, Te7 and Te8 coincide very well with each other, and, as the electronic density of Cs and Te is similar, do not harm the refinement model significantly.

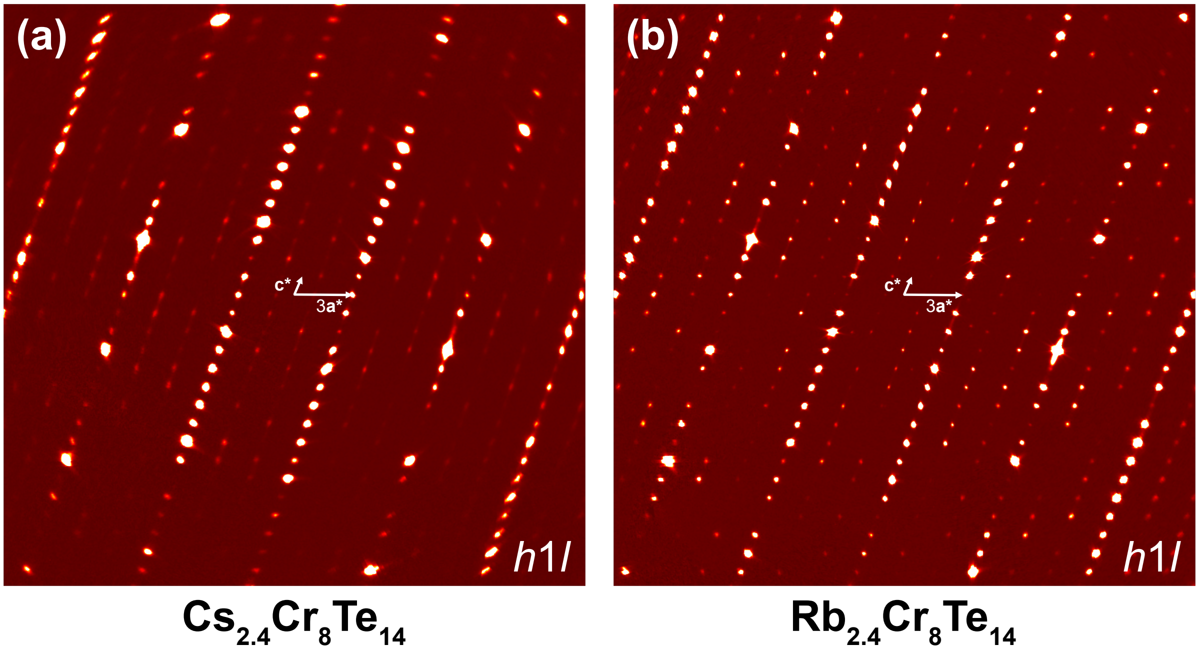

Figure 1: Reconstrcted reciprocal *h*1*l* planes of (a) $Cs_{2.4}Cr_8Te_{14}$ and (b) $Rb_{2.4}Cr_8Te_{14}$

$Rb_{2.4}Cr_8Te_{14}$

In the diffraction pattern and refinement of $Rb_{2.4}Cr_8Te_{14}$, diffuse scattering and stacking disorder appear to be less of a problem. However, the diffraction pattern is polluted by traces of residual Rb_2Te_3 flux stuck at the crystal plates. This additional phase systematically

influences all reflections with $h = 10n, k = 0, l = 7m$ $(m, n \in \mathbf{Z})$. If the second phase is ignored during integration, reflections of this group all have significantly higher F^2_{obs} values compared to F^2_{calc}. Using the multicrystal option of CrysalisPro, all overlapping reflections can be eliminated from the obtained intensity file. However, the refinement R-values do not significantly improve compared to when just omitting the most intense difference reflections from a one-domain integration.

Elemental composition maps

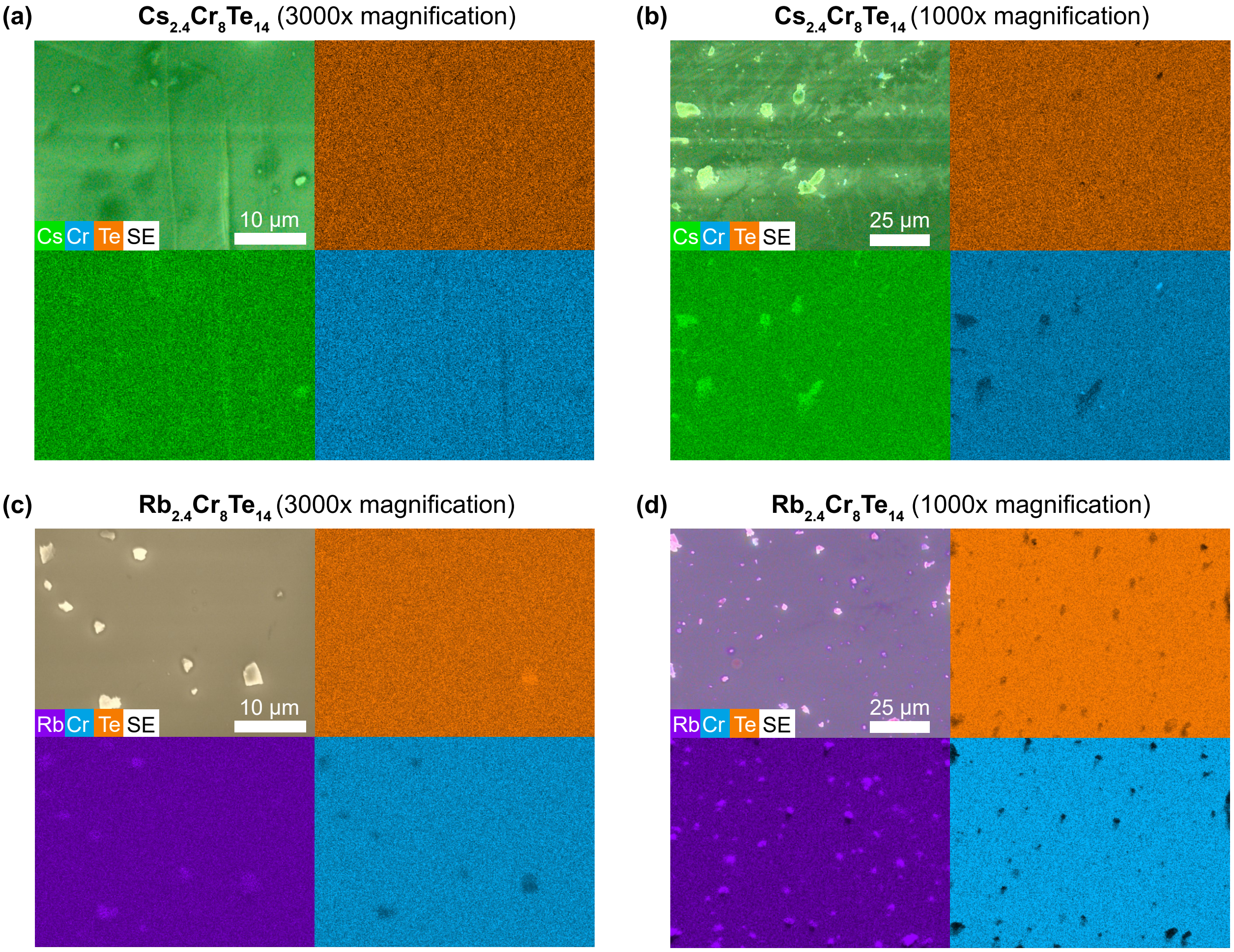

Figure 2: EDS maps of $Cs_{2.4}Cr_8Te_{14}$ at (a) 3000× magnification and (b) 1000× magnification as well as $Rb_{2.4}Cr_8Te_{14}$ at (c) 3000× magnification and (d) 1000× magnification. Elemental maps are depicted as overlays on the secondary electron (SE) image.

Table 4: Individual calculated elemental compositions of $Cs_{2.4}Cr_8Te_{14}$, $Rb_{2.4}Cr_8Te_{14}$, and $CsCr_5Te_8$ including standard deviations using EDS with specification of the batch, the crystal and the site it was measured at.

$Cs_{2.4}Cr_8Te_{14}$	
Location	Elemental composition
batch 1, crystal 1, site 1	$Cs_{2.64(9)}Cr_8Te_{14.16(26)}$
batch 1, crystal 1, site 2	$Cs_{2.40(4)}Cr_8Te_{13.89(7)}$
batch 1, crystal 2, site 1	$Cs_{3.13(24)}Cr_8Te_{15.5(6)}$
batch 1, crystal 2, site 2	$Cs_{2.64(5)}Cr_8Te_{13.79(17)}$
batch 2, crystal 1, site 1	$Cs_{2.84(9)}Cr_8Te_{14.52(21)}$
batch 2, crystal 1, site 2	$Cs_{2.70(10)}Cr_8Te_{14.14(21)}$
batch 2, crystal 2, site 1	$Cs_{3.10(22)}Cr_8Te_{14.8(5)}$
batch 2, crystal 2, site 2	$Cs_{3.44(20)}Cr_8Te_{15.7(6)}$
$Rb_{2.4}Cr_8Te_{14}$	
Location	Elemental composition
batch 1, crystal 1, site 1	$Rb_{2.35(17)}Cr_8Te_{14.1(3)}$
batch 1, crystal 1, site 2	$Rb_{2.49(6)}Cr_8Te_{13.80(11)}$
batch 1, crystal 2, site 1	$Rb_{2.62(25)}Cr_8Te_{13.52(22)}$
batch 1, crystal 2, site 2	$Rb_{2.58(23)}Cr_8Te_{13.71(24)}$
batch 2, crystal 1, site 1	$Rb_{2.66(6)}Cr_8Te_{13.39(7)}$
batch 2, crystal 1, site 2	$Rb_{2.80(6)}Cr_8Te_{13.38(9)}$
batch 2, crystal 2, site 1	$Rb_{2.51(17)}Cr_8Te_{13.58(17)}$
batch 2, crystal 2, site 2	$Rb_{2.43(10)}Cr_8Te_{13.66(11)}$
$CsCr_5Te_8$	
Location	Elemental composition
batch 1, crystal 1, site 1	$Cs_{1.12(10)}Cr_5Te_{8.54(17)}$
batch 1, crystal 1, site 2	$Cs_{1.14(13)}Cr_5Te_{8.45(15)}$
batch 1, crystal 2, site 1	$Cs_{1.05(13)}Cr_5Te_{7.81(19)}$
batch 1, crystal 2, site 2	$Cs_{0.95(7)}Cr_5Te_{7.80(7)}$
batch 2, crystal 1, site 1	$Cs_{0.94(4)}Cr_5Te_{7.89(10)}$
batch 2, crystal 1, site 2	$Cs_{0.96(5)}Cr_5Te_{8.03(6)}$
batch 2, crystal 2, site 1	$Cs_{1.01(6)}Cr_5Te_{7.98(19)}$
batch 2, crystal 2, site 2	$Cs_{0.95(2)}Cr_5Te_{7.92(5)}$

Detailed magnetic characterization

$Cs_{2.4}Cr_8Te_{14}$

The temperature-dependent measurements of the magnetic moment of $Cs_{2.4}Cr_8Te_{14}$ with the external field applied parallel and perpendicular to the *ab* plane, which are depicted in Figure

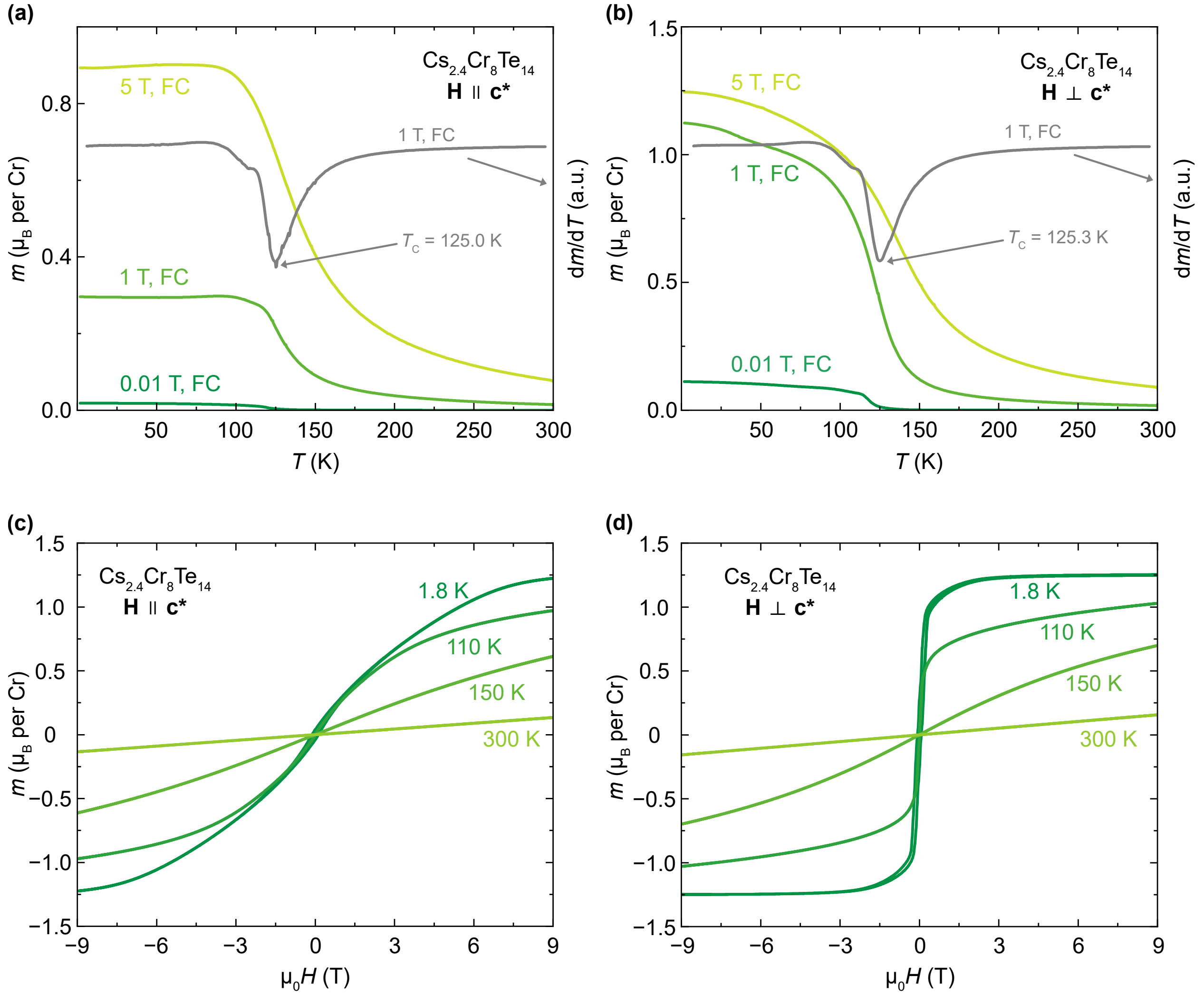

Figure 3: Temperature-dependent (a,b) and field-dependent (c,d) magnetic moment of $Cs_{2.4}Cr_8Te_{14}$. Temperature-dependent field-cooled magnetic moment of $Cs_{2.4}Cr_8Te_{14}$ between 1.8 K and 300 T at 1 T and 5 T parallel to the **c** axis, (a), and perpendicular to it, (b) First derivatives of the magnetic moment against temperature are depicted in grey. Field-dependent measurements of the magnetic moment of $Cs_{2.4}Cr_8Te_{14}$ between −9 T and 9 T parallel to the **c** axis, (c), and perpendicular to it, (d)

3(a,b). ZFC and FC measurements match each other with fields applied down to 1 T, as shown in Figure 4. The ferrimagnetic transition temperature can be estimated based on the derivative of the temperature-dependent measurements of the magnetic moment depicted in 3(a,b). The field-dependent magnetization measurements of $Cs_{2.4}Cr_8Te_{14}$ are presented in Figure 3(c,d) for $T = 1.8$ K, 110 K, 150 K, and 300 K with the external magnetic field parallel and perpendicular to the c axis respectively. The measurements show the presence of a

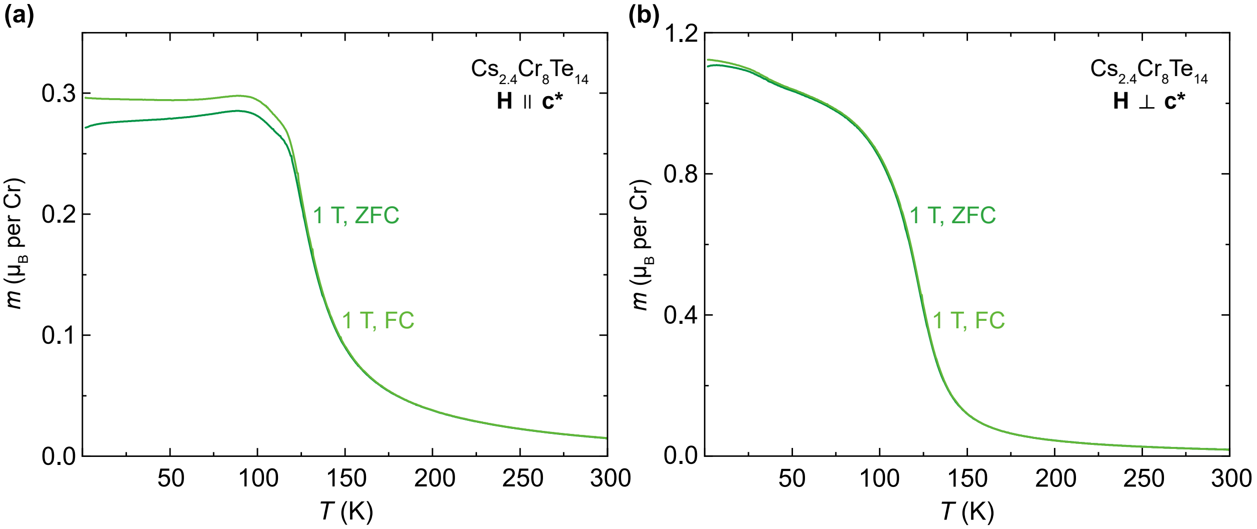

Figure 4: Temperature-dependent measurement of the magnetic moment of $Cs_{2.4}Cr_8Te_{14}$ with an applied field of 1 T along the hard axis, (a), and easy plane, (b) in zero-field-cooling (ZFC) and field-cooling (FC) mode.

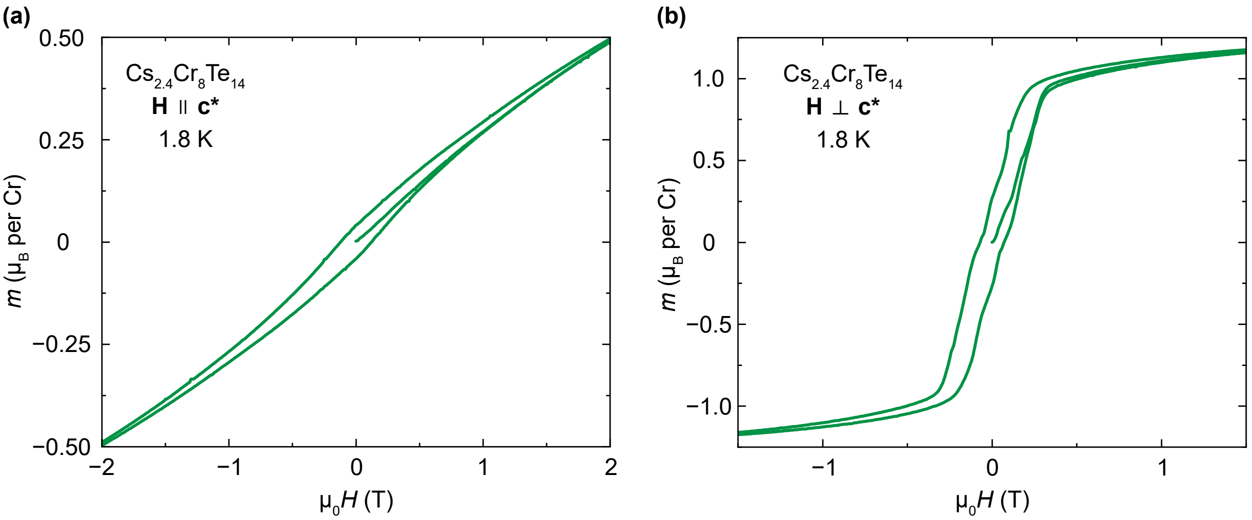

Figure 5: Enlarged representation of the hysteresis loops in measurements of the field-dependent magnetic moment of $Cs_{2.4}Cr_8Te_{14}$ with fields applied (a) parallel to **c*** and (b) perpendicular to **c***.

hysteresis loop, depicted enlarged in Figure 5. For both $\mathbf{H} \parallel \mathbf{c*} = 9\,\mathrm{T}$ and $\mathbf{H} \perp \mathbf{c*} = 9\,\mathrm{T}$ the magnetic moment saturates at about $m = 1.24\,\mu_B/\mathrm{Cr}$.

$Rb_{2.4}Cr_8Te_{14}$

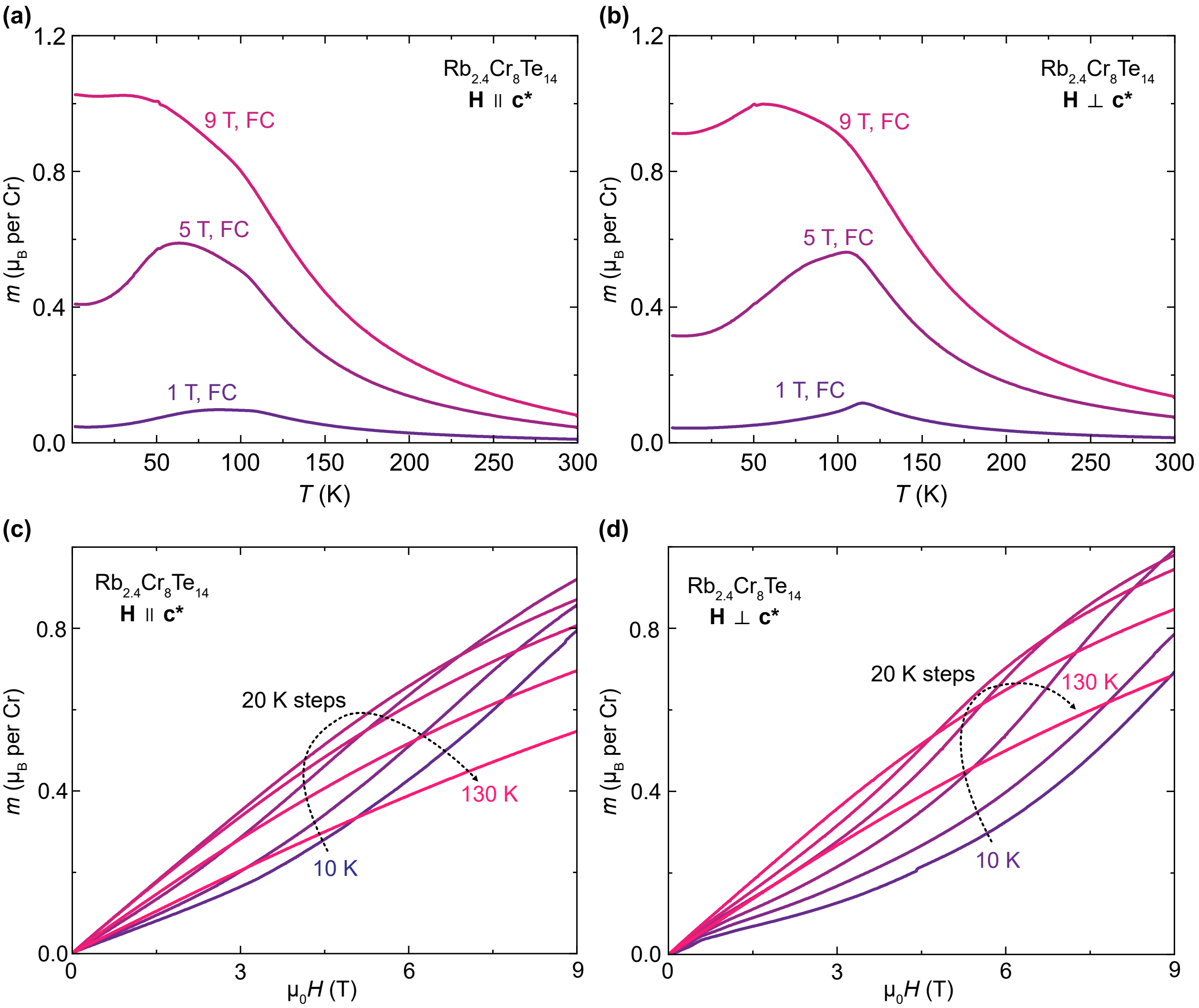

Figure 6: Temperature-dependent (a,b) and field-dependent (c,d) magnetization of $Rb_{2.4}Cr_8Te_{14}$. Temperature-dependent field-cooled magnetization of $Rb_{2.4}Cr_8Te_{14}$ between 1.8 K and 300 K at 1 T and 5 T parallel to the **c** axis, (a), and perpendicular to it, (b). Field-dependent measurements of the magnetic moment of $Rb_{2.4}Cr_8Te_{14}$ between −9 T and 9 T parallel to the **c** axis, (c), and perpendicular to it, (d)

Temperature-dependent measurements of the magnetic moment of $Rb_{2.4}Cr_8Te_{14}$ with the external field applied parallel and perpendicular to the ab plane are illustrated in Figure 6(a,b). For an applied field of 1 T, the $m(T)$ shows the characteristic peak shape of an antiferromagnet with a maximum reached at the Néel temperature $T_{\mathrm{N}} = 114.5\,\mathrm{K}$. While this maximum is rather sharp for $\mu_0 H = 1\,\mathrm{T}$ and $\mathbf{H} \perp \mathbf{c*}$, for $\mathbf{H} \parallel \mathbf{c*}$, the maximum is

broadened. This effect becomes increasingly stronger for both orientations when increasing the applied field. For $\mu_0 H = 9\,\mathrm{T}$ with $\mathbf{H} \parallel \mathbf{c*}$ the magnetic moment saturates at about $1\,\mu_B/\mathrm{Cr}$, which likely represents a shift to ferromagnetism. For $\mu_0 H = 9\,\mathrm{T}$ with $\mathbf{H} \perp \mathbf{c*}$ this transition to ferromagnetism is incomplete, and the sample shows a strong widening around the maximum in magnetization.

Figure 6(c,d) show the magnetic moment for applied fields between 0 T and 9 T at temperatures between 10 K and 130 K in steps of 20 K with fields applied $\mathbf{H} \perp \mathbf{c*}$ and $\mathbf{H} \parallel \mathbf{c*}$ respectively. Other than for $Cs_{2.4}Cr_8Te_{14}$, no saturation was observed up to fields of 9 T in either crystal orientation. The change of curvature appears at different temperatures, approximately around 100 K for $\mathbf{H} \perp \mathbf{c*}$, while already at ca. 60 K for $\mathbf{H} \parallel \mathbf{c*}$.

$CsCr_5Te_8$

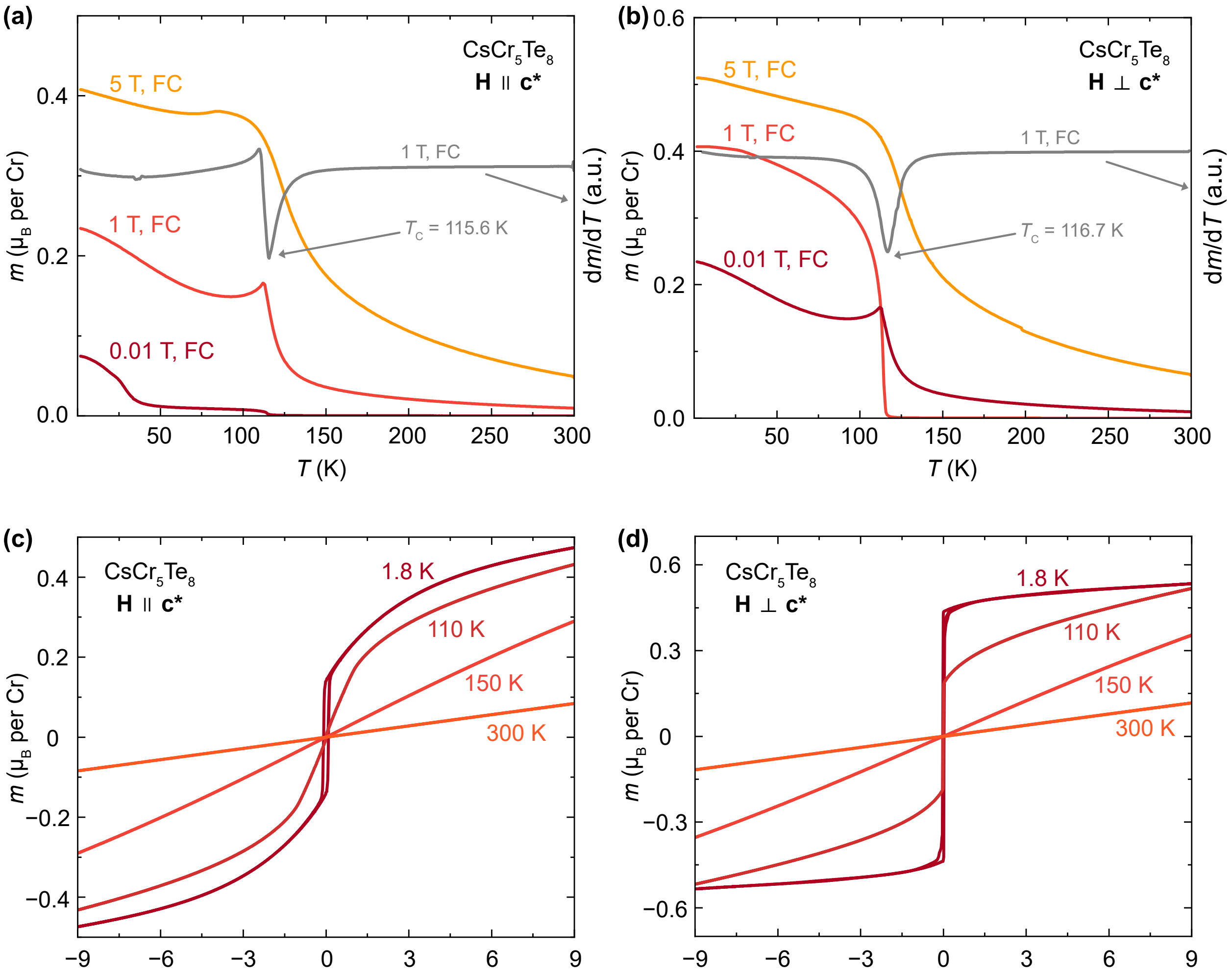

Figure 7: Temperature-dependent (a,b) and field-dependent (c,d) magnetic moment of $CsCr_5Te_8$. Temperature-dependent field-cooled magnetization of $CsCr_5Te_8$ between 1.8 K and 300 T at 0.01 T, 1 T, 5 T parallel to the **c** axis, (a), and perpendicular to it (b). First derivatives of the magnetic moment against temperature are depicted in grey. Field-dependent measurements of the magnetic moment of $CsCr_5Te_8$ between −9 T and 9 T parallel to the **c** axis, (c), and perpendicular to it (d).

Previous investigations of the magnetic properties of $CsCr_5Te_8$ were limited to polycrystalline samples,[2] possibly due to smaller crystal sizes resulting from different synthesis conditions. Our self-flux growth method yielded crystals large enough for direction-dependent measurements of the magnetic moment. Plots of $m(H)$ in $\mathbf{H} \parallel \mathbf{c^*}$ (Figure 7(a)) and $\mathbf{H} \perp \mathbf{c^*}$ (Figure 7(b)) orientations show a ferrimagnetic transition at $T_C = 115.6$ K. This transition is

especially pronounced at the higher measured magnetic fields of $\mu_0 H = 1\,\mathrm{T}$ and $5\,\mathrm{T}$) and is at a slightly lower temperature than the previously reported T_C of $125\,\mathrm{K}$.[2] For a very small field ($0.01\,\mathrm{T}$), especially with $\mathbf{H} \parallel \mathbf{c^*}$ a second transition around $T \approx 28\,\mathrm{K}$ becomes apparent. A similar behavior in other layered magnetic Cr-based materials has been attributed to an additional low temperature magnetic order.[3,4] Magnetic measurements on powder $CsCr_5Te_8$ by Yamazaki *et al.* also shows an increase of the magnetic moment below the T_C suggesting an intrinsic mechanism. While the observed magnetic moment for $\mathbf{H} \parallel \mathbf{c^*}$ is strongly dependent on the applied field, for $\mathbf{H} \perp \mathbf{c^*}$ the magnetic moment is more similar, hinting at an easy plane within the **ab** plane. This is confirmed by the $m(H)$ measurements depicted in Figure 7(c) and (d). For $\mathbf{H} \parallel \mathbf{c^*}$, a small hysteresis loop with a coercivity of $\approx |900\,\mathrm{Oe}|$ and a magnetic moment, which does not saturate for fields up to $9\,\mathrm{T}$ is observed. For $\mathbf{H} \perp \mathbf{c^*}$, corresponding to the easy plane, the magnetic moment reaches a plateau at around 0.5 μ_B/Cr for $\mu_0 H > 1000\,\mathrm{Oe}$. A small continuous increase at higher fields is likely caused by paramagnetic impurities, likely Cs_2Te_3.

Powder diffraction patterns

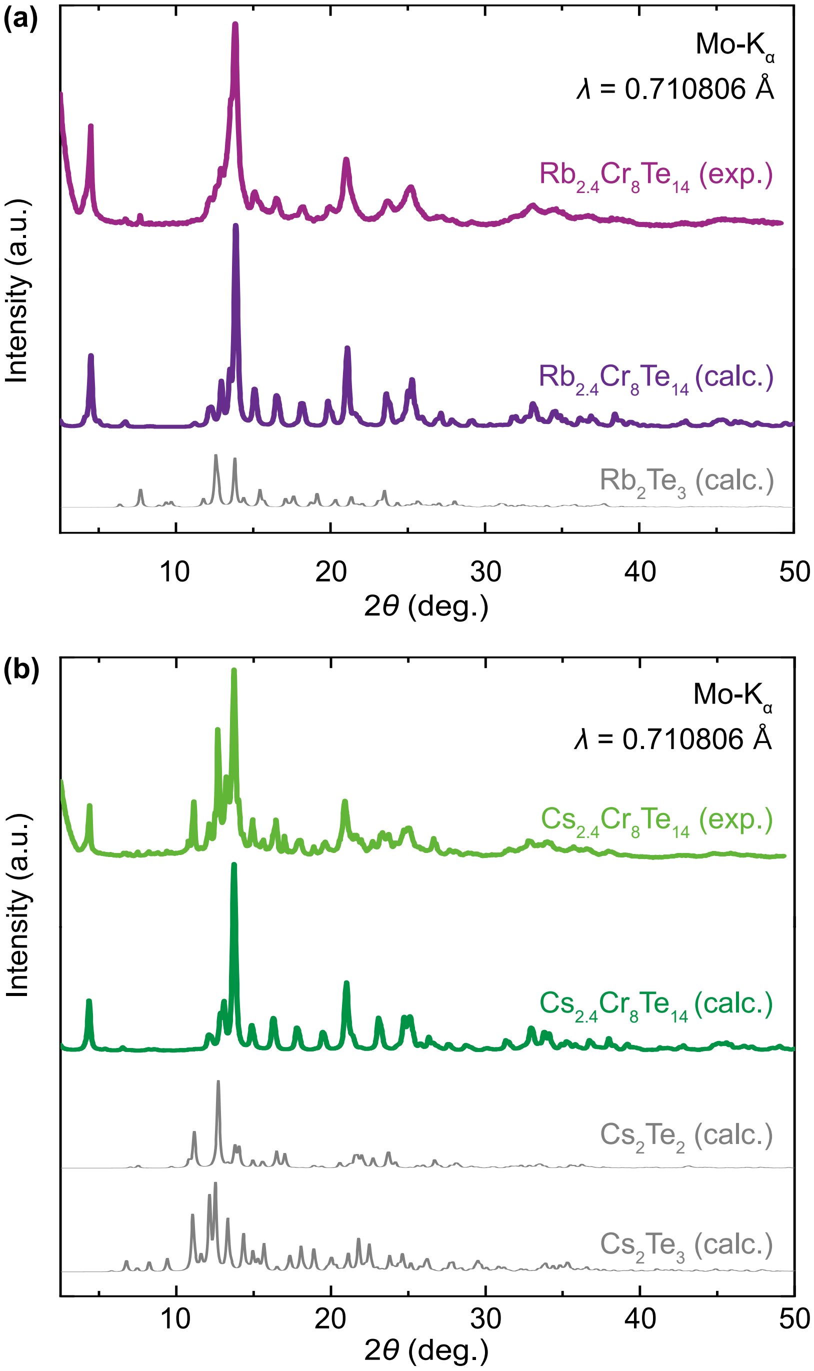

Figure 8: Comparison of the experimental (exp.) PXRD patterns crushed crystals of (a) $Rb_{2.4}Cr_8Te_{14}$ and (b) $Cs_{2.4}Cr_8Te_{14}$ with calculated patterns of $Rb_{2.4}Cr_8Te_{14}$, $Cs_{2.4}Cr_8Te_{14}$ and the respective side phases Rb_2Te_3 or Cs_2Te_3 and Cs_2Te_2.

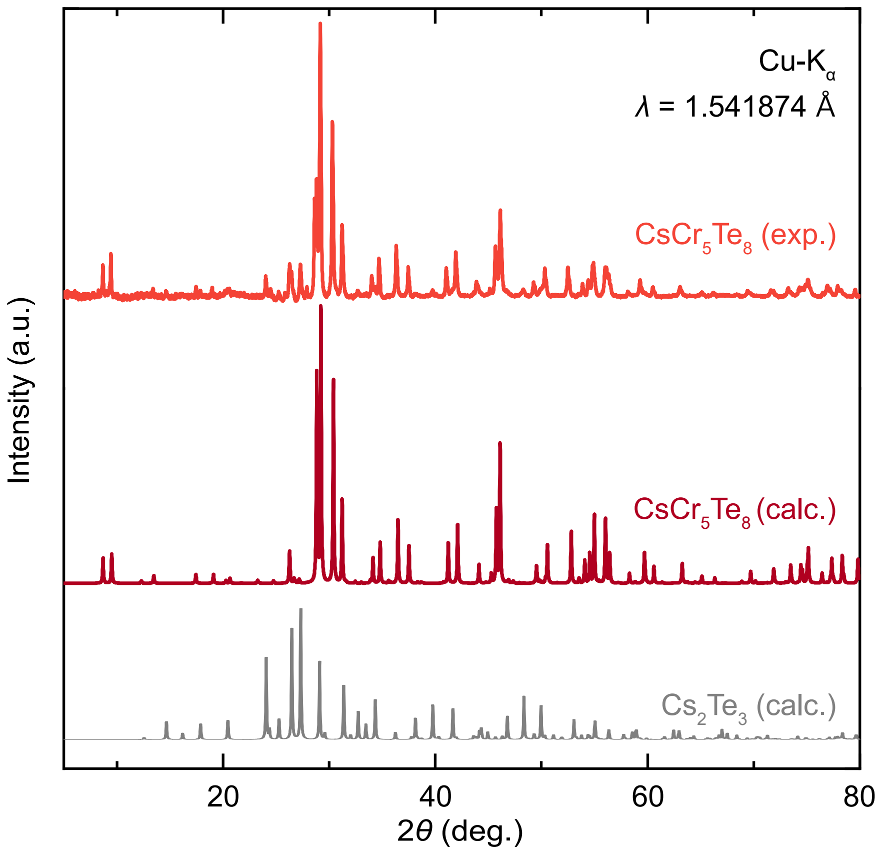

Figure 9: Comparison of the experimental (exp.) PXRD pattern of crushed $CsCr_5Te_8$ crystals with calculated patterns of $CsCr_5Te_8$ and the side phases Cs_2Te_3.

References

(1) de la Flor, G.; Orobengoa, D.; Tasci, E.; Perez-Mato, J. M.; Aroyo, M. I. Comparison of structures applying the tools available at the Bilbao Crystallographic Server. *Journal of Applied Crystallography* **2016**, *49*, 653–664.

(2) Yamazaki, S.; Ueda, Y. New Ferromagnetic Chromium Chalcogenides, ACr5Te8 (A = K, Cs and Rb). Solid Compounds of Transition Elements I. 2011; pp 17–20.

(3) Witteveen, C.; Nocerino, E.; López-Paz, S. A.; Jeschke, H. O.; Pomjakushin, V. Y.; Månsson, M.; von Rohr, F. O. Synthesis and anisotropic magnetic properties of $LiCrTe_2$ single crystals with a triangular-lattice antiferromagnetic structure. *Journal of Physics: Materials* **2023**, *6*, 035001.

(4) López-Paz, S. A.; Guguchia, Z.; Pomjakushin, V. Y.; Witteveen, C.; Cervellino, A.; Luetkens, H.; Casati, N.; Morpurgo, A. F.; von Rohr, F. O. Dynamic magnetic crossover at the origin of the hidden-order in van der Waals antiferromagnet CrSBr. *Nature Communications* **2022**, *13*, 4745.
